# Endothelin B receptors exert antipruritic effects via peripheral κ-opioid receptors

**DOI:** 10.3892/etm.2012.624

**Published:** 2012-06-27

**Authors:** WENJIN JI, JIEXIAN LIANG, ZHIWEI ZHANG

**Affiliations:** 1 Departments of Anesthesiology, and; 2 Pediatric Cardiology, Guangdong Cardiovascular Institute, Guangdong General Hospital, Guangdong Academy of Medical Sciences;; 3 Postgraduate Institute, Southern Medical University, Guangzhou, P.R. China

**Keywords:** itch, κ-opioid receptor, naloxone, nor-Binaltorphimine, endothelin-1

## Abstract

Endothelin B receptor agonists exert antipruritic effects on itching induced via endothelin-1 (ET-1) and compound 48/80. Peripheral µ- and κ-opioid receptors (MORs and KORs, respectively) are reported to be involved in the anti-nociceptive properties triggered by ET_B_ agonists. Therefore, we investigated the role of peripheral opioid receptors in the scratching response induced by ET-1. ET_A_ and ET_B_ antagonists and non-selective and selective opioid receptor antagonists were co-injected with ET-1 in the neck of mice and the number of scratching bouts was counted. Pretreatment with systemically administered naloxone significantly reduced the number of scratches, while co-injection of naloxone substantially augmented the effect of ET-1. Co-injection of nor-Binaltorphimine (nor-BNI), a KOR antagonist, significantly increased the number of scratches induced by ET-1. However, CTOP (a MOR antagonist) and naltrindole [a δ-opioid receptor (DOR) antagonist] did not alter the scratching response elicited by ET-1. These results indicate that peripheral KORs mediate the antipruritic effect of endothelin B receptor activation.

## Introduction

Endothelin-1 (ET-1) is generated by a variety of cell types, including endothelial cells, vascular smooth muscle cells, leukocytes, cardiomyocytes, mesangial cells, certain tumor cell lines, as well as neurons and glia in both the central and peripheral nervous systems (1–3). Local administration of ET-1 induces nociceptive behaviors in animals (4,5) and causes pain in humans (6). ET-1 also elicits pruritus in mice (7–9) and humans (6,10). The signaling of ET-1 is mediated by two main membrane G-protein coupled receptor subtypes, ET_A_ and ET_B_ (11). Blockade of the ET_A_ receptor inhibits the scratching response induced by ET-1 (7,8,12), while co-injection of ET_B_ antagonists increases scratching bouts induced by ET-1 (8,12). By contrast, ET_B_ agonists exert antipruritic effects of itch induced by both ET-1 and compound 48/80 (8).

ET-1 is secreted in response to inflammation, tissue injury and other stress stimuli. Therefore, ET_B_ is a promising target to mitigate ET-1-induced itch symptoms. The mechanisms that drive the antipruritic effects of ET_B_ remain to be elucidated. It has been reported that keratinocytes express ET_B_ receptors, and upon activation may lead to the release of opium-like substances (13). Peripheral µ- and κ-opioid receptors (MORs and KORs, respectively) are reported to be involved in the anti-nociception effect triggered by ET_B_ agonists (13,14) Peripheral δ-opioid receptors (DORs) are also considered to be involved in the antinociceptive activity of opioid peptides, which are released from neutrophils and in response to endothelin-A receptor antagonists (15,16).

Peripheral MORs and KORs, but not DORs, may play key roles in pruritus (17,18). For example, topical application of naltrexone inhibits pruritus in patients with atopic dermatitis (19). In addition, ICI 204448, a peripherally restricted KOR agonist, was found to antagonize chloroquine-induced scratching in mice (20). We note that neuron excitability is reduced upon opioid receptor activation, which is due to the inhibition of voltage-dependent Ca^2+^ channels and adenyl cyclase, as well as the activation of K^+^ channels (21). Notably, SQ22536, a selective inhibitor of adenyl cyclase, inhibits the scratching response induced by ET-1 (12). Therefore, our goal was to investigate the effects of opioid receptor antagonists on the scratching response induced by ET-1.

## Materials and methods

### Animals

Male C57BL/6J mice, weighing 20–22 g, were obtained from the Center for Laboratory Animals, Sun Yat-Sen University (Guangzhou, China). The animals were housed at room temperature (22±1°C) on a 12/12-h light (8 a.m.-8 p.m.)/dark (8 p.m.-8 a.m.) cycle and had free access to rodent food and water. The experimental procedures and the animal use and care protocols were approved by the Committee on Ethical Use of Animals of Guangdong General Hospital (Guangzhou, China). Our procedures also followed the National Institutes of Health’s animal use and care guidelines. All efforts were made to minimize animal suffering and to reduce the number of animals used.

### Drugs and chemicals

Synthetic ET-1, BQ-123 (ET_A_ antagonist) and BQ-788 (ET_B_ antagonist) were purchased from American Peptides (Sunnyvale, CA, USA). Naloxone hydro-chloride was supplied by Kawin Technology Share-Holding Co. (Beijing, China). CTOP (Phe-Cys-Tyr-Trp-Orn-Thr-Pen-Thr-NH_2_; MOR antagonist) and nor-Binaltorphimine dihydrochloride (nor-BNI; KOR antagonist) were purchased from Sigma Chemical Co. (St. Louis, MO, USA). Naltrindole hydrochloride (DOR antagonist) was purchased from Tocris (Bristol, UK). All drugs were diluted in phosphate-buffered saline (PBS) and the pH was adjusted to 7.4.

### Pruritus model and behavioral analysis

The pruritus model was established as previously described (12). Briefly, at one day after shaving the rostral part of the back of the neck, mice were placed into a small plastic chamber (22×12×20 cm^3^) 30 min prior to the experiment. For drug administration, mice were briefly removed from the chamber and 50 μl of each test drug was intradermally injected with a 30-gauge needle (detailed description of injection has been described elsewhere) (22). The mice were then returned to the chamber and hind limb scratching that was directed towards the shaved area at the back of the neck was observed and recorded for 30 min. One scratch was defined as a lift of the hind limb towards the injection site and then a reposition of the limb back to the floor, regardless of the scratching strokes that took place between the two movements. All testing was conducted between 10:00 and 16:00. To reduce the injection times, antagonists or inhibitors (BQ-123 50 nmol, BQ-788 15 nmol, naloxone 2 nmol or 0.5 mg/kg, CTOP 10 nmol, nor-BNI 5 nmol, naltrindole 60 nmol) were co-injected with ET-1 (50 pmol) in a volume of 50 μl. Naloxone (2 nmol or 0.5 mg/kg) was subcutaneously injected 15 min prior to the administration of 50 μl ET-1 (50 pmol) to investigate the role of systemic naloxone administration. The specific doses of BQ-123, BQ-788, naloxone, CTOP and naltrindole used were selected based on previous studies (12,13,15,23).

### Statistical analysis

Minitab 16 for windows (Minitab Inc., State College, PA, USA) was used for statistical analysis. All results are expressed as means ± SEM. Data were statistically evaluated by analysis of variance followed by Bonferroni’s test or, when only two means were to be compared, unpaired Student’s t-test. P<0.05 was considered to indicate a statistically significant result.

## Results

### Intradermal injections

Intradermal injections of PBS, BQ-123, BQ-788, naloxone, CTOP or naltrindole caused no noticeable scratching response. By contrast, a scratching response was elicited by intradermal injection of ET-1, or a high concentration of nor-BNI. However, there was no noticeable increment of hoarsening, grooming or paw licking with intradermal ET-1 or nor-BNI injections.

### BQ-123 and ET-1 injection

**Figure 1:**
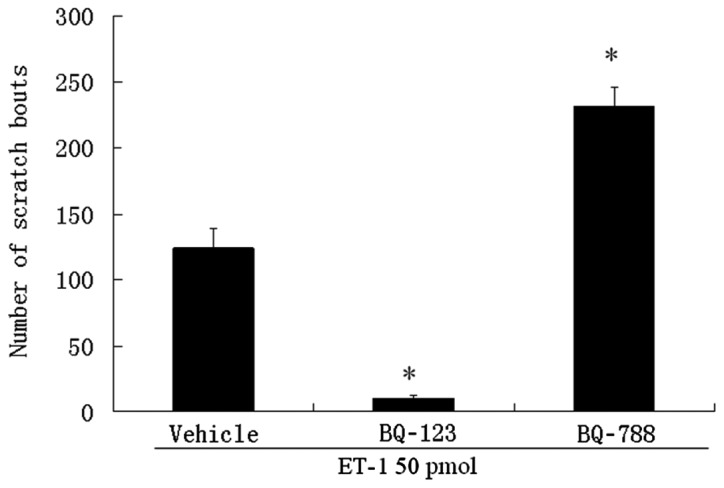
Effects of specific ET_A_ and ET_B_ receptor antagonists on the scratching response induced by ET-1. A total of 50 pmol ET-1 only, or together with 50 nmol BQ-123 (ET_A_ antagonist), or 15 nmol BQ-788 (ET_B_ antagonist), were injected intradermally (50 μl). Each bar indicates the number of bouts of scratching for 30 min following the injection of ET-1. Data are expressed as mean ± SEM and n=10 in each treatment group. ^*^P<0.05, relative to the group treated with only ET-1. ET-1, endothelin-1.

Intradermal injection of 50 pmol ET-1 evoked scratching bouts (124±14 bouts). Scratching bouts induced by ET-1 were markedly inhibited when they were co-administered locally with the selective ET_A_ receptor antagonist BQ-123 (Fig. 1). In contrast, local co-injection of the selective ET_B_ receptor antagonist BQ-788, substantially augmented the effect of ET-1 (231±14 bouts; Fig. 1).

### Naloxone and ET-1

**Figure 2:**
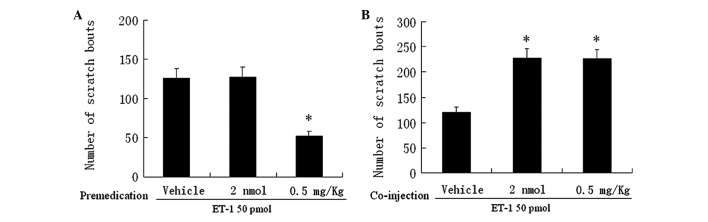
Effects of premedication or co-injection with naloxone on the scratching response induced by ET-1. (A) Subcutaneous naloxone (2 nmol or 0.5 mg/kg) or PBS (vehicle) was systemically injected 15 min prior to the administration of ET-1 (50 pmol in 50 μl PBS). (B) Naloxone (2 nmol or 0.5 mg/kg) or PBS was co-injected with ET-1 (50 pmol in 50 μl PBS). Each bar indicates the scratch bouts for 30 min following the injection of ET-1. Data are expressed as mean ± SEM, and n=8 in each treatment group. ^*^P<0.05 as compared with the group treated with only ET-1. ET-1, endothelin-1.

We then examined the effects of naloxone, an antagonist of opioid receptors, on the scratching response induced by ET-1. Pretreatment with systemically administered naloxone 0.5 mg/kg significantly reduced the number of scratches to 52±6 compared to that induced following pretreatment with PBS (126±12, Fig. 2A). Co-injection of naloxone 2 nmol or 0.5 mg/kg substantially and similarly augmented the effect of ET-1 (Fig. 2B). Pretreatment with a low dose of naloxone (2 nmol), however, was not able to alter the scratching response (Fig. 2A). Thus, local, but not systemic naloxone, prevented the antipruritic effect induced by activation of the ET_B_ receptor, suggesting the involvement of peripheral opioid receptors in pruritis.

### CTOP, nor-BNI, naltrindole and ET-1

**Figure 3:**
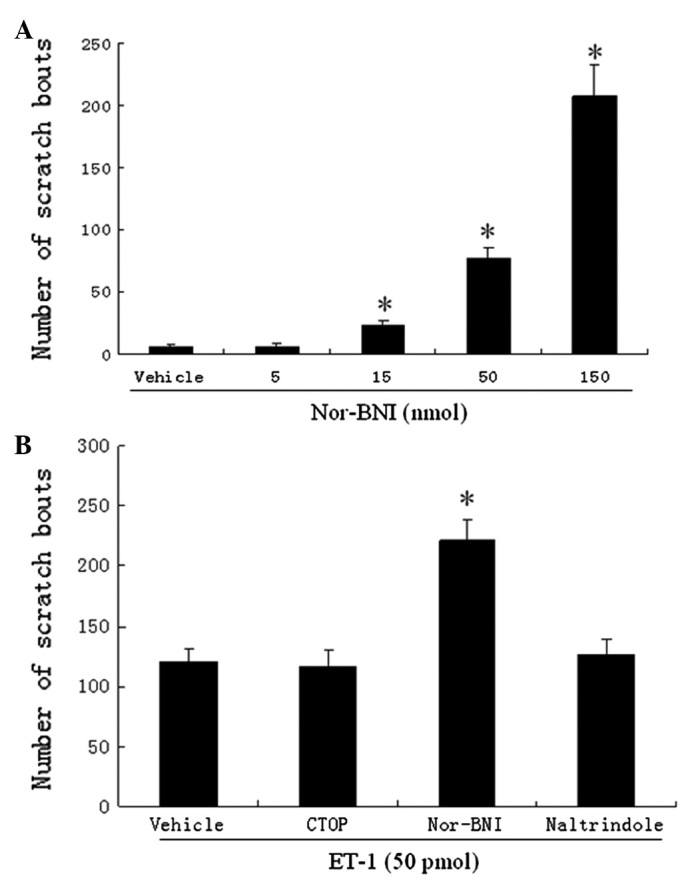
Pruritus induced by nor-Binaltorphimine dihydrochloride (nor-BNI) and effects of selective opioid receptor antagonists on the scratching response induced by ET-1. (A) Nor-BNI at various doses was intradermally injected in 50 μl PBS. (B) CTOP 10 nmol, nor-BNI 5 nmol, naltrindole 60 nmol or PBS was co-injected with ET-1 (50 pmol in 50 μl PBS). Data are expressed as mean ± SEM, and n=8 in each treatment group. ^*^P<0.05, as compared with the group treated with vehicle. ET-1, endothelin-1.

We also examined the effects of CTOP, nor-BNI and naltrindole (MOR, KOR and DOR antagonists, respectively) on the scratching response induced by ET-1. Intradermal injections of nor-BNI evoked dose-dependent scratching bouts (Fig. 3A). We note that 5 nmol of nor-BNI was selected since it did not cause a noticeable scratching response. Co-injection of nor-BNI significantly increased the number of scratches (from an average of 121 to 222 bouts; Fig. 3B) induced by ET-1. CTOP and naltrindole did not alter the scratching response to ET-1 (Fig. 3B).

## Discussion

In the present study, we demonstrated that the activation of peripheral endothelin B receptors exerts antipruritic effects via peripheral KORs. For example, co-injection of naloxone (a non-selective opioid receptor antagonist) and nor-BNI (a KOR antagonist) with ET-1 significantly increased the number of scratches, while co-injection of CTOP (a MOR antagonist) and naltrindole (a DOR antagonist) with ET-1 did not alter the scratching response. Notably the doses of CTOP and naltrin-dole are sufficient to reduce the analgesic effects evoked by endogenous opioid peptides (13,15).

The opioid system plays a pivotal role in modulating pruritus (18). Opioid-induced pruritus is a well-known side effect of postoperative analgesia attributed to spinal or epidural morphine, as well as administration of other MOR agonists (24,25). Notably, MOR antagonists and KOR agonists have been reported to treat pruritus effectively in patients with chronic renal failure, cholestasis and atopic dermatitis (26). Opioid receptors are expressed in peripheral nerve endings and keratinocytes in human skin (27). The gastrin-releasing peptide receptor (GRPR) is an itch-specific molecule in the spinal cord (28). For example, ablation of lamina I neurons expressing GRPR in the spinal cord of mice produces profound scratching deficits in response to itching (pruritogenic) stimuli (29). Notably, GRP is co-localized with MOR in mouse skin (30). These data suggest that the opioid receptors involved in itching sensations are peripheral, as well as central (18).

Naloxone has been shown to inhibit scratching induced both by capsaicin in inflamed skin and by several pruritogens (23,31–33). It is noteworthy that co-injection with naloxone increased scratches induced by ET-1. Intradermal or subcutaneous injections of KOR antagonists induced scratching responses both in this study and in previous research (34), while intradermal injections of naloxone alone did not induce scratching, suggesting that the blockade of MORs may inhibit the pruritogenic effect of KOR antagonists.

Loperamide, a peripherally restricted MOR agonist, was also found to antagonize scratching evoked by compound 48/80 in mice (35). However, scratching responses were evoked by intradermally injected morphine, fentanyl and loperamide (36). Naloxone methiodide, a peripherally restricted opioid receptor antagonist, significantly suppressed scratching behavior induced by MOR agonists loperamide and DAMGO (37). It has also been demonstrated that ET_B_ agonists injected into the neck induced scratching in female BALB/C mice (7), while no scratching response was induced in male Swiss mice (8). By contrast, ET_B_ antagonists injected into the neck induced scratching in male Swiss mice (8), while it did not induce scratching in this study, as well as previous studies using male C57BL/6J mice (12). CTOP was not able to inhibit the scratching response induced by ET-1 in the current study, indicating the significance of genetic backgrounds in determining puritogenic sensitivity to peripheral MOR agonists.

Activation of neuronal KORs reduces neuronal excitability via various pathways (21). However, it is possible to exert antipruritic effects via non-neuronal pathways. For example, we recently observed that activation of transient receptor potential ankyrin subfamily member 1 (TRPA1) suppresses itch responses induced by ET-1 (38). Notably, TRPA1 and KORs are expressed in human keratinocytes (39,40). It will be valuable to evaluate the role of TRPA1 and KORs in keratinocytes.

In summary, we investigated the involvement of peripheral opioid receptors in ET-1-induced pruritus in mice. We suggest that peripheral KORs mediate the antipruritic effects of endothelin B receptor activation.
